# Exploring the Bedouin Syndrome in the Football Fan Culture: Addressing the Hooliganism Phenomena through Networks of Violent Behavior

**DOI:** 10.3390/ijerph19159711

**Published:** 2022-08-06

**Authors:** Thyago Celso Cavalcante Nepomuceno, Victor Diogho Heuer de Carvalho, Lúcio Camara e Silva, Jadielson Alves de Moura, Ana Paula Cabral Seixas Costa

**Affiliations:** 1 Núcleo de Tecnologia, Federal University of Pernambuco, Caruaru 55014-900, Brazil; 2 Campus do Sertão, Federal University of Alagoas, Delmiro Gouveia 57480-000, Brazil; 3 Departamento de Engenharia de Produção, Federal University of Pernambuco, Recife 50670-901, Brazil

**Keywords:** behavioral sports economics, violent behavior, hooliganism, Bedouin syndrome, network analysis

## Abstract

The Bedouin syndrome represents social interactions based on four premises: a friend of my friend is my friend, a friend of my enemy is my enemy, an enemy of my friend is my enemy, and an enemy of my enemy is my friend. These extensive associations exist in many social and economic relationships, such as market competition, neighborhood relations, political behavior, student gangs, organized crime, and the violent behavior of sports spectators (hooliganism) worldwide. This work tests the Bedouin syndrome hypothesis considering the violent behavior in the football fan culture. We construct relational networks of social affinities to represent the social interactions of organized fan bases (*Torcidas organizadas*) involved in hooligan violence in Pernambuco, Brazil. Contrary to prior expectations, the results evidence no statistical support for the Bedouin syndrome in 13 of the 15 analyzed clubs. There is weak statistical support in two interactions and strong statistical support in one interaction to state that a friend of my enemy is my friend (instead of an enemy). The only support for the Bedouin syndrome is circumstantial based on a prior assumption of an alliance. We propose a network development that can be more suitable to represent football fans’ violent behavior. The results contribute to understanding the hooliganism social phenomenon in football-rooted cultures and their impact on public health, identifying potential determinants for organized violence by young spectators’ and supporting police strategies by defining relevance scores for the most potential clashes and coalitions of gangs.

## 1. Introduction

Hooliganism can be defined as the violent behavior of sports spectators, both in groups and alone [1,2,3]. The modern usage of the term dates back to the United Kingdom hooligan gangs in the late 50s and during the 60s; groups of young supporters of Chelsea, Millwall, Liverpool, and Everton engaged in crowd violence, public disorder, and several train-wrecking incidents, although episodes of riot, vandalism and violent disorder from sports fans can be recognized much before [4]. Since the 60s, football (known as soccer in the United States) violence has been an increasing concern for many European countries, recently stated by many reports on the escalating number of football-related offenses, arrests, and the propensity to the unruly behavior of young people [5]. According to Spaaij [6], situational, environmental, and social factors unrelated to the sporting event are determinants for this form of collective aggression.

This is one of the most recurrent social problems faced by football-rooted cultures in Latin America, such as Brazil, Argentina, Uruguay, Chile, Peru, Mexico, and Colombia. According to Brandão et al. [7], Brazil has the highest number of hooligan-related deaths in the world. The author states that the culture of violence in football is something all countries have in common. However, Brazil’s hooliganism has become more intense due to social aggressiveness and political corruption [8].

Most of the violence is attributed to the so-called *Torcidas Organizadas*, football firms, or fan bases—groups of football fans supporting the same team—that emerged in the late 60s from the *Torcidas Uniformizadas*, which implemented a new dynamic for supporting the clubs and interpersonal relationships through their members [9]. During the 70s, there was a clear differentiation between the ordinary supporter and the “organized” one (belonging to a football firm) at football matches [10]. Today in Brazil, football matches, especially derbies (having clubs from the same city), require a great effort from the public authorities to allocate officers, technology, and resources to maintain a safe atmosphere preventing the clash of gangs around the stadiums.

Nepomuceno et al. [11] tested the hypothesis that alcohol is one of those situational determinants of violent behavior by football fans by assessing Pernambuco’s State Law 13748/2009. This public sanction prohibited the selling and intake of alcoholic beverages in the stadiums during official soccer matches (i.e., state, regional, national and international championships) from April 2009 to December 2015 (except during the 2014 World Cup). Their results were supported by the Pernambuco State Legislature, which abolished the law in 2016. Since the publication, three other Brazilian states have used their work and results in Legislative plenaries and forums to discuss the issue. Two of them (Alagoas and Ceará) have followed Pernambuco’s decision to abolish the sanction.

From Nepomuceno et al.’s [11] perspective, contextual factors determining the relative importance of the match, such as commemorative days, the club’s rank position, prior provocations, and derbies, play an essential role in the short-run determination of the aggressive behavior by the fans. This perspective is also shared by Spaaij [6] and Collins [12], arguing that spontaneous episodes of collective violence appear to be related to events on the playing field because fans are subject to the same rhythms of dramatic tension as players. Nevertheless, despite these relevant contributions, quantitative inferences are still scarce measures in supporting policing strategies to increase the community’s security by evaluating trends and potential determinants of football gang’s aggressiveness in multiple perspectives, engaging tools, and methodologies to better understand this reality.

A different perspective on sports spectators’ aggressive behavior was conceptualized in Paul Harrison [13] as social interactions within disputing football gangs named the Bedouin syndrome. This paper aims to provide a methodology for constructing and analyzing football supporter networks’ violent behavior based on the Bedouin syndrome. The syndrome relies on four deterministic assertions that represent a cause-and-effect relationship among the social groups: the friend of my friend is my friend; the friend of my enemy is my enemy, the enemy of my friend is my enemy, and the enemy of my enemy is my friend.

The sequence of this article is divided as follows: Section 2 presents the theoretical background with concepts and some previous research about sports spectators’ behaviors and the Bedouin syndrome; Section 3 is dedicated to presenting the applied methodology; Section 4 contains the results of the methodology application and a discussion about them; Section 5 continues presenting the obtained results, but with a specific focus on the networks visualizations; Section 6 concludes the article.

## 2. Sports Spectators’ Behavior

The rivalry of fans of different sports is a vector for violent behavior, as in the case of hooliganism. The literature presented throughout this section highlights studies aimed at understanding this social phenomenon. Such context can be understood as the result of a highly positive feeling for a particular team, leading to a passionate and loyal follow-up, as well as the feeling of hatred for other teams, especially those that, sportingly, can be understood as common opponents or rivals [14,15,16].

Guo et al. [17] proposed and applied a method for mining the psychological factors of sports fans. Using a questionnaire, they used fan community members as subjects to investigate the factors influencing aggressive behavior among fans. They applied the K-means method improved with principal component analysis (PCA) for clustering the psychological factors, discovering that there are three main factors triggering violence according to the fan community: (i) modern social pressure, regarding the accelerated pace of life and the increasing competition which put people under pressure; (ii) increasing in fan appreciation, related to the closest ways that fans obtained to follow the games involving their teams; and (iii) management factors, related to the quality of organizational management and improper maintenance of order in the matches.

The research by van Ham et al. [2] aims to improve the understanding of the extent to which mutually organized clashes differ from ‘regular’ clashes between fans, seeking to fit this with existing theories of violence in football. Data were collected from different sources through questionnaires, interviews, case studies, comparison of cases, and analysis of offenders, identifying behavioral indicators exploring the existence of significant differences in the relationship between violent acts in sports and measures of psychological traits using t and chi-square tests. The results indicated that antagonistic relationships between hooligan groups are not prerequisites for mutually arranged confrontational events. These events seem to revolve predominantly around establishing or maintaining a collective reputation that demonstrates strength, seeking excitement, violent behavior, and establishing or maintaining social dominance.

However, not all studies are focused on the violent aspects of the interaction between groups of rival sports fans, as is the case discussed by Joern and Havelund [18]. Their study focused on the behavior of Danish Ultras, who have been playing a positive and proactive role in disseminating a culture rejecting discrimination and violence among sports fans. This demonstrates how broad the social spectrum of sports spectator behavior is, ranging from one extreme where there is intense rivalry, which often culminates in physical aggression (and even culminating in gun violence [19]), to the other extreme, which works in an anti-violence culture, in favor of pacifism and healthy interaction among sports fans.

The following section presents the violent confrontation between sports fans from the perspective of Bedouin theory, also considering an approach from social networks.

### The Bedouin Syndrome and Network

The Bedouin syndrome has been claimed to be one of the critical social interactions and territorial determinants of youth violence among sports spectators worldwide [20]. These social interactions are composed of alliance and rivalry strategies from the gang members about their potential opponents. Especially in European and Latin American countries with deep football-rooted cultures, the problem immerges into a significant issue of public safety policies for young people, which compose most gang members in organized crime. According to Junger-Tas [21], these juveniles fail to obtain rewards by conventional subsystems. They turn to other marginal and deviant groups to be rewarded and recognized by behaving such as a “tough” person committing offenses.

Most of the empirical evidence converges to Russell’s [22] statement about the temporality of the hooligan Bedouin association, i.e., a dynamic instead of the deterministic relationship among sports opponents leading friends to become enemies depending on contextual or environmental changes. If the Bedouin syndrome creates friendly relations among young people, it generates aggressive hostilities and clashes among those supporters of different backgrounds [20]. The Bedouin syndrome concept is crucial for policymakers to understand juvenile delinquency’s social interactions, motivators, and consequences and to guarantee a safe urban atmosphere through coherent public policies. To the best of our knowledge, quantitative inferences to test and construct preliminary evidence of the Bedouin syndrome have never been adequately implemented. The discussions found in the scientific literature are limited to theoretical contributions to hooliganism’s social constructs, lacking support for police strategies to prevent criminal and aggressive behavior.

The design of empirical network representations about the incidence of violence among sports spectators based on the Bedouin syndrome can be a viable way to generate an understanding of how fan bases are structured and interrelate, being able to guarantee to the authorities a preview of which “coalitions” of supporters may or may not be harmful to public welfare. Besides supporting fast decision making, Bedouin networks’ design has relevant statistical properties that may help policymakers predict the most probable clash of gangs in the urban space and guarantee public security [11]. Such an approach is in continuously grown over the past decades, providing applications in the fields of information technology, engineering, management, economics, industrial organization, behavioral sciences, and policy modeling [23,24,25,26]. According to Coles [27], there is a common sense that the analysis of social networks contributes positively to the identification of structural patterns in criminal conduct and facilitates faster decision-making. Some examples are found in Sarnecki’s [28] work, which employed network analysis methods to assess the social ties, bonds, and relations among 22,000 youth suspected of about 29,000 felonies and misdemeanors in Stockholm, and in the networks of drug offenders discussed by Heber [29].

Due to the capacity to construct third-party alliances and rivalries representations, the Bedouin syndrome concepts can help the social comprehension of hooliganism through the design and analysis of the network of aggressive behavior [20,30]. Potential violence incidents among sports spectators may be abstracted and predicted without additional information on whether they are direct friends or adversaries [22]. In this work, instead of a deterministic relationship, we propose a social network structure where the affinities among the football gangs are concomitants and independent of each other, meaning that they can co-occur for a pair of groups regardless of the association between each of them with a third group.

The main contribution of this network representation is to allow a broader representation of the Bedouin syndrome because two friends may present a mutual enemy, not just as a consequence of a friendship [20]. In addition, in the case of a network designed under simultaneous affinities, internal determinants as described by Dunning et al. [4] such as the age propensity to unruly behavior, sexual segregation, ethnic and social-economic characteristics, opportunities, and, in certain instances, the territorial unity may appear to be more relevant in the long term to describe the violent behavior of sports spectators. The design of edges and nodes representing the violence and interaction among the club gangs in this work is made, resorting to the same dataset provided by Nepomuceno et al. [11] on the hooligan incidents in Pernambuco over the past decade.

## 3. Data and Methodology

We test the Bedouin syndrome hypothesis on the assumed rivalry relations among Brazilian *torcidas organizadas* (organized fan bases). The analytic network frames the existing violent interaction of supporters from three important football clubs (clubs which we call “heads”) in Brazil’s northeast region, and their interactions with other Brazilian football clubs’ supporters. These interactions represent the fans’ violent behavior in official matches (local, regional, national and international championships) [19,31]. Each of the three head clubs has more the one organized fan base. The crowd violence is ignited to different proportions with different opponents or specific seasons [2,6,10,15].

**Figure 1:**
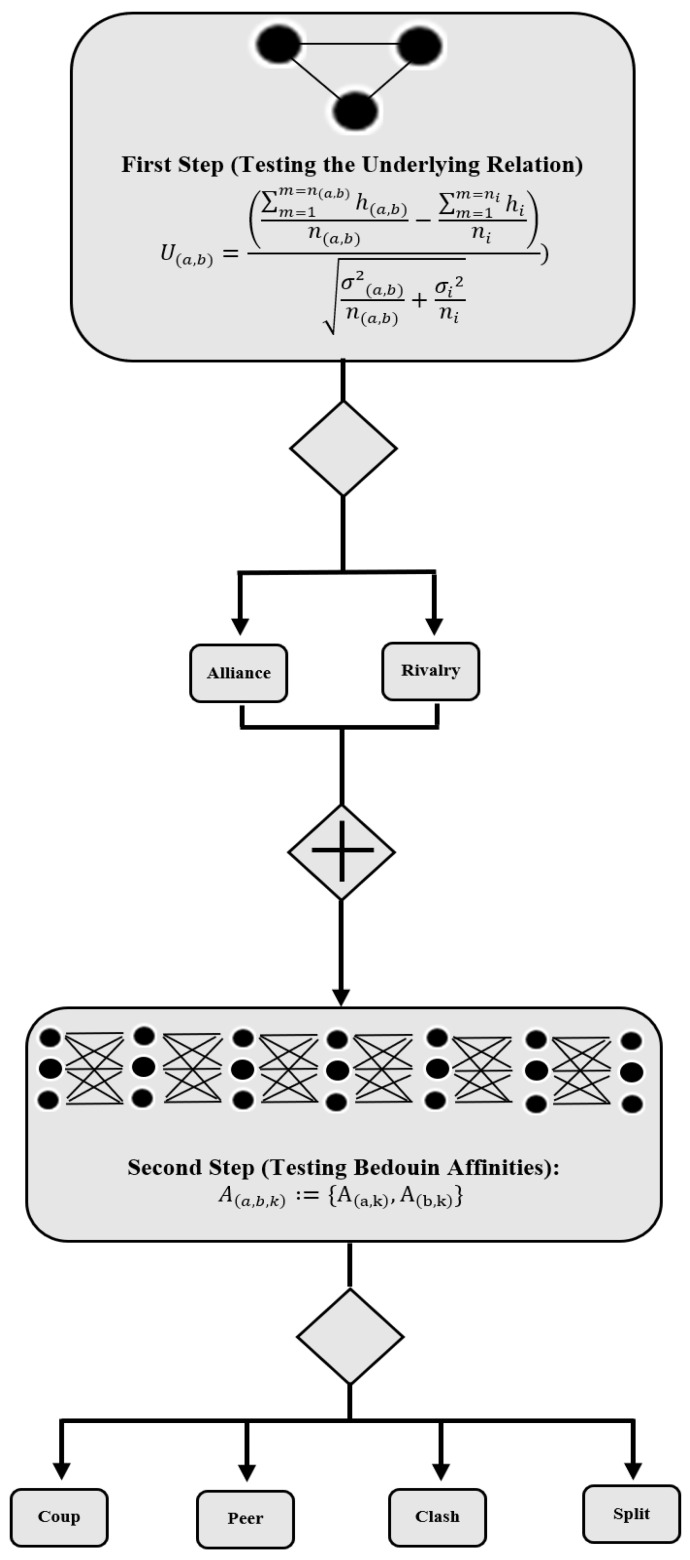
Flow diagram representing the methodology steps.

The methodology for testing the Bedouin syndrome comprises two steps with multiple hypothesis tests. In the first step, we aim to determine whether two heads declared enemies (or declared friends) are, in fact, enemies (or friends) based on the mean distribution of their hooligan incidents. Hooligan incidents are the criminal occurrences (incidents) recorded by the police related to violence, such as promoting turmoil, threat, slander, assault, and battery, among others. The result of these tests for each pair of heads a and b defines an underlying relation “$U_{\left(a,b\right)}$” of alliance or rivalry. After determining the relations among the heads, the second step aims at testing whether similar relations are maintained for a third club declaring to be an enemy or friend of one of the heads. We define an affinity relation “$A_{\left(h\right)}$” for each h combination of two heads with a third club. The hypothesis tests in the second step can result in a coup, clash, peer, or split affinity associations. This methodology is illustrated in the diagram in Figure 1.

For a complete panorama, the network visualizations are constructed with all associations instead of only the statistical relevant. For simplification, instead of considering particular fan bases in the network, the violent incidents are aggregated into the related clubs to reduce the exhaustive and many-sided network interactions and to avoid additional statistical computation. The nodes in the landscape visualization in Section 5 represent the groups of hooligan incidents by supporters of specific clubs instead of each fan base. However, there is no direct association or support from the clubs to the episodes of violence.

### 3.1. Data and Network Tools

Different criminology theories, such as the Theory of Rational Choice of Gary Becker [32], the theory of routine activity of Cohen and Felson [33] and the Ostrom [34] and Nepomuceno et al. [35,36] framework of crime determination highlight different perspectives for different types of crimes [37]. Thus, we limited our analysis to aggression-based data and misdemeanors where the offenders are motivated by a desire to commit the violent approach, and the victim is irrelevant (opportunistic victims). The police collected a total of 1363 hooligan occurrences in 374 football matches. In the original dataset, many occurrences were related to property offenses, drug traffic, and other misdemeanors that clearly have a different perspective from the one we are addressing in this work. Those were removed from the analysis.

The data concerns the violent behavior of football supporters during the period from February 2005 to August 2015. All incidents from less aggravated misdemeanors such as promoting turmoil, threat, slander, lewd acts, false alarm, or breaking into the field or restricted area, to more severe felonies such as assault and battery, aggravated battery, contempt of cop, fisticuffs, incitement to crime, damage to public or private property, violent disorder and affray and unlawful use or attack on means of transportation were considered.

Only matches settled in the state of Pernambuco with one of the three heads (namely Sport Club Recife, Santa Cruz FC, and Clube Náutico Capibaribe) are taken into consideration to build the network of affinities described in Table 1. For this reason, the network visualization of the violent behavior in the Section 5 of this work reports edges that connect the several Brazilian football clubs with at least one of the Pernambuco’s three head clubs, but no connections among themselves.

**Figure 2:**
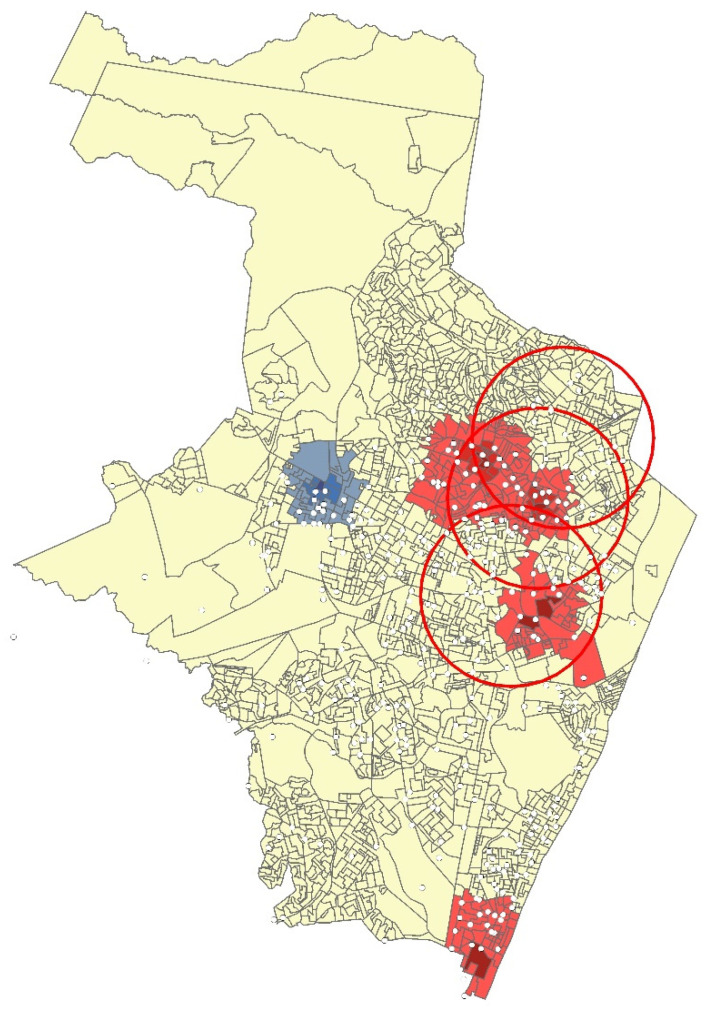
Distribution of incidents.

Recife (Pernambuco capital) is the terrace for about 90% of the hooligan incidents in the dataset. Most of the city’s hooligan incidents are concentrated up to 3 km from the stadiums during the sports event, as illustrated in Figure 2 with some of the occurrences for one specific season (data obtained from Nepomuceno and Costa [38]). Most of the hotspot concentration of incidents (red areas) are near one of the three stadiums (inside the red circles). However, one neighborhood concentrates incidents relatively distant from the stadiums (Boa Viagem), representing the crime capillarity throughout the city.

The methodology used to construct and visualize the hooligan network associations in this work is based on the scientometrics rezoning present in most of the tools that portray bibliometric landscapes, such as the software VOSviewer [39], Gephi [40,41], and CiteSpace [42]. The clustering of the clubs in terms of network affinity is based on statistical hypothesis tests. In this work, the program package Pajek [43,44,45] is used to design the social network analysis of the supporter violent behavior in terms of nodes and edges connections. A similar visualization can be observed in the Nepomuceno et al. [11] assessment of alcohol sanctions within football stadiums. The association of nodes (clubs) is obtained by identifying relational affinities consisting of concentration patterns from pairwise interactions among the evaluated items, i.e., interaction between two head clubs and another club.

The edges between two node structures in the hooligan network visualization represent this interaction. Besides designating whenever there is an association, they also indicate the strength of this association [46]. The node size and the length of the edges are defined based on the number of hooligan occurrences each head presents with one club and their number of connections i.e., clubs, whereas at least one hooligan incident occurs. Each node position in the network also has a significant meaning; it defines the grade of rivalry or alliances from one club to another. Four types of Bedouin simultaneous affinities among the clubs can be decomposed from these rivalry or alliance relations described in Table 1.

According to Kessler [47], in a scientific network, two items form a coup when a third is connected to both of them. In the hooligan social networks, two friendly head clubs (as the items in this representation) form a coup with a third club whenever the latter presents hooligan occurrences with both of them (i.e., whenever there is an edge in the schematic representation that connects two friends with an adversary). Peer affinities in social networks are interpreted as symmetric relations, whereas each pair of instances lists each other as friends [48]. Forms of clashes (opponent groups or individuals with a mutual enemy) and splits (opponent groups or individuals with a mutual friend) affinities are decomposed in the structural balance of social network settings [49].

### 3.2. First Step: Testing the Underlying Relation

The first two affinities can be associated with the Bedouin syndrome in the reasoning presented in Table 2.

The football fan bases in Brazil have associated friends (called *Toricidas Aliadas* or *Torcidas Coligadas*) and declared enemies (*Torcidas Rivais*). Testing the Bedouin syndrome is the same as determining if two declared enemies (or declared friends) are enemies (or friends) based on the distribution of their incidents. See if similar relations are maintained for the third group of declared enemies or friends. For this purpose, an underlying relation $U_{\left(a,b\right)}$ of alliance or rivalry is defined for each (*a*,*b*) pair combination of heads and an affinity relation $A_{\left(h\right)}$ for each h combination of two heads with one club. The underlying and affinity relations are developed as a set of hypothesis tests on the mean distribution of the incidents to state the friendship level of two heads (alliance or rivalry), and their association with a third group (coup, clash, peer, split). In the first case, assuming unequal variances/samples, the underlying relationship is determined by the difference between the hooligan means among the heads and a benchmark value, which is the sample mean of the overall hooligan incidents among the clubs:

(1) $$U_{\left(a,b\right)}=\frac{\left(\frac{\sum_{m=1}^{m=n_{\left(a,b\right)}}h_{\left(a,b\right)}}{n_{\left(a,b\right)}}-\frac{\sum_{m=1}^{m=n_{i}}h_{i}}{n_{i}}\right)}{\sqrt{\frac{\sigma 2_{\left(a,b\right)}}{n_{\left(a,b\right)}}+\frac{\sigma_{i}{}^{2}}{n_{i}}}}$$

Resorting to ($n_{\left(a,b\right)}-1)$ + ($n_{i}-1)$ degrees of freedom to determine the association, where:

$h_{\left(a,b\right)}$ represents the incidents in matches where heads *a* and *b* are competing;

$h_{i}$ represents the total number of hooligan occurrences for all *i* pair of clubs (including the heads);

$n_{\left(a,b\right)}$ represents the number of matches where *a* and *b* are competing with each other;

$n_{i}$ represents the total number of matches for all *i* pair of clubs (including the heads);

$\sigma_{\left(a,b\right)}$ represents the standard deviation of the hooligan incidents in the matches which the head clubs *a* and *b* are competing with each other, and

$\sigma_{i}$ represents the standard deviation of the hooligan incidents for all *i* pair of clubs (including the heads).

Whenever the mean of the heads’ incidents $h_{\left(a,b\right)}$ is different (bigger) than the mean of hooligan incidents for all pairs of clubs $h_{i}$, the underlying relation ($U_{\left(a,b\right)}$ is enough to reject the null hypothesis that the mean distribution of incidents between a and b is statically similar to the mean distribution of the overall incidents for each combination of clubs. If this is the case, an underlying rivalry relation is identified, i.e., the two head clubs in this pairwise comparison are enemies because the mean of their incidents is statistically bigger than the expected mean value for the hooliganism. Suppose the mean of the heads’ occurrences is different (smaller) than the mean of hooligan incidents for all pairs of clubs. In that case, the underlying relation rejects the null hypothesis for the similarity in the mean distribution of incidents, i.e., the two heads in the comparison are friends because their mean hooligan incident is smaller than the expected value for the hooliganism among the clubs. Otherwise, whenever the value of the head’s underlying relation is not sufficient to support the null hypothesis rejection, the hooligan distribution from the heads a and b are statistically equal to the overall mean distribution of hooligan incidents. In this case, an undefined relation is identified.

### 3.3. Second Step: Testing the Bedouin Affinities

After determining the relations among the heads (alliance or rivalry), the second step to test the Bedouin syndrome is to perform an additional test on the remaining clubs to determine their associations with each head (coup, clash, peer, split). Similarly, assuming unequal variances, a set of hypothesis tests is performed to compare the mean distribution of incidents with a benchmark value (the overall mean of occurrences) in order to identify affinities relations:

(2) $$A_{\left(a,b,k\right)}=\{A_{\left(a,k\right)}=\frac{\left(\frac{\sum_{m=1}^{m=n_{\left(a,k\right)}}h_{\left(a,k\right)}}{n_{\left(a,k\right)}}-\frac{\sum_{m=1}^{m=n}h_{i}}{n}\right)}{\sqrt{\frac{\sigma 2_{\left(a,k\right)}}{n_{\left(a,k\right)}}+\frac{\sigma_{i}{}^{2}}{n_{i}}}},A_{\left(b,k\right)}=\frac{\left(\frac{\sum_{m=1}^{m=n_{\left(b,k\right)}}h_{\left(b,k\right)}}{n_{\left(b,k\right)}}-\frac{\sum_{m=1}^{m=n}h_{i}}{n_{i}}\right)}{\sqrt{\frac{\sigma 2_{\left(b,k\right)}}{n_{\left(b,k\right)}}+\frac{\sigma_{i}{}^{2}}{n_{i}}}}\}$$

where the affinity relation $A_{\left(a,b,k\right)}$ between the two heads *a* and *b* and the club *k* is defined to have the values from the set consisting of both the relations between the head a and club *k* and head *b* and club *k* interactions, i.e., whether each pair (*a*, *k*) and (*b*, *k*) are given as friends or enemies. A coup affinity, for instance, must have the heads (*a*, *b*) as friends ($U_{\left(a,b\right)}$ statistic fails to reject the null hypothesis), the head a as an enemy of club *k* and the head b as an enemy of club *k* (both $A_{\left(a,k\right)}$ and $A_{\left(b,k\right)}$ statistics supporting the rejection of the null hypothesis).

## 4. Results and Discussion

Initially, the Bedouin syndrome is tested on the declared alliances made by the football fan clubs, using the total number of violent events for the club from February 2005 to August 2015, avoiding sided networks, as stated in Section 3 about the data and methodology. Each fan base declares a relationship with other fan bases as friends or enemies. We aim to test whether the declared relationship is sufficient to support the Bedouin syndrome on third parties (fan bases). Following the two-step methodology from the last section, the underlying relation is tested concerning the three head clubs in Pernambuco to verify whether they are friends or enemies.

Then, the Bedouin affinities are tested to verify a third club (fan base) as a friend of my friend, friend of my enemy, an enemy of my friend, or an enemy of my enemy relationship. Section 5 provides a general representation of the underlying relationships among the three head clubs in Pernambuco to express the set of clubs with a higher probability of an incident of violence occurring with the related head. In addition, two instances of affinities are designed in three network landscapes to bring tangible considerations regarding the importance of this identification for public safety decision making.

### 4.1. Testing the Underlying Relation

The set of declared friends for each head is presented in Table A1 in the Appendix A. The organized fan bases have used social media and websites to promote their actions before and after the sports events and to declare alliances with other fan bases of different regions. These combinations into alliances or coalitions as the “friends side” and enemies or rivals as the “enemies side” are the bases for testing the Bedouin syndrome effect over the relationship networks among the fan bases. Therefore, they have a widely known old relationship of declared alliance and rivalry with no need for deep investigation.

The *p*-values for testing the underlying relations among the three heads are described in Table 3. The aggregate standard deviation, including all the heads’ occurrences, is 15.463 for an average of 8.064 incidents per match. We considered the heterogeneity in the variance distribution in the incidents between the Torcida Jovem (Sport Club Recife) and Torcida Inferno Coral (Santa Cruz FC), and between Torcida Fanáutico (Clube Náutico Capibaribe) and Torcida Inferno Coral (Santa Cruz FC). The highest difference in the standard deviations (aggregate and pairs) is for the incidents from the Torcida Inferno Coral (Santa Cruz FC) and Torcida Fanáutico (Clube Náutico Capibaribe).

Torcida Jovem Sport has an underlying relation of a rivalry with both Torcida Inferno Coral and Torcida Fanáutico. The comparisons between the Torcida Inferno Coral and Torcida Fanáutico, however, fail to reject the null hypothesis of equality in the mean distribution of hooligan incidents, meaning an undefined underlying relation with respect to Santa Cruz FC and Náutico Capibaribe, which can mean rivalry, alliance, or none, depending on the context. Despite the declared rivalry between these clubs from the same city in derbies, the number of hooligan incidents around their matches is close to the hooliganism’s expected value considering a typical match. For this reason, it does not have statistical support for the assumption of rivalry due to the low average of incidents in the analyzed period.

### 4.2. Testing the Bedouin Affinities

Based on each head of underlying relations (Table 3), we can define what relation should be expected with a third-party fan base testing Bedouin affinities’ assumption. Table A1 presents the alliances each head declares to have with other Brazilian football firms (second and third columns) and which affinity should be expected of this alliance (fourth column) compared to one of the heads (first column). Table 4 brings the results of testing the Bedouin syndrome. According to Equation (2), the mean distribution of hooligan incidents for each match disputed by the heads and the clubs in Table 4 is compared with a benchmark value (in this case, the overall mean of occurrences) in order to identify the affinities relations.

Under the assumption of unequal variances, there is no evidence to support the Bedouin syndrome in 13 of the 15 analyzed clubs. Results that are significant at a *p*-value < 0.1 are highlighted in bold. This is justified to report that if we relax the typical adopted confidence level from 0.05 to 0.1, we would not only lack evidence to support the Bedouin syndrome but also report some (weak) statistical support to the contrary. The weak statistical support (0.1 > *p*-value > 0.05) for alliances in two interactions (Sport Recife vs. Ypiranga and Náutico Capibaribe vs. Cruzeiro EC) and strong support (*p*-value < 0.05) for alliances in one interaction (Sport Recife vs. SE Palmeiras) lead to the conclusion (based on the declared alliances and rivalry of Table A1) that a friend of my enemy is my friend (instead of an enemy). Strong support for the Bedouin syndrome regarding a peer affinity is found in the fan interactions of Náutico Capibaribe vs. Atlético MG if we consider Santa Cruz FC building an alliance underlying relation with Náutico Capibaribe (see Table 3 and related explanations). Lastly, we can find some weak statistical support for the Bedouin syndrome only in Santa Cruz FC vs. SC Internacional interactions. These first results on testing differences in the mean distribution of the hooliganism on the declared alliances do not provide significant statistical support for the Bedouin syndrome for most disputed matches.

## 5. Network Visualization

**Figure 3:**
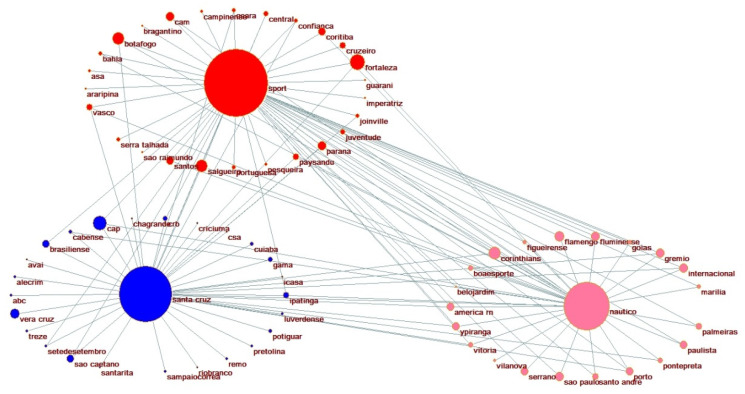
Hooligan network clusters by clubs.

Testing differences in the mean distribution of hooliganism on the declared alliances does not provide significant statistical evidence to support the Bedouin syndrome. We aim to evaluate and construct a broader network of aggressive behavior considering all the matches where incidents of violence have occurred. The hooligan network pictured in Figure 3 presents all possible hostile associations identified in the dataset. The circular clustering representations are constructed with the support of Pajek 5.01 layout tools, using the partitions defined by the underlying and affinities relations values described in the methodology to allocate each club as enemies and using two complete optimized layers in x-direction [50].

Whenever an incident of violence occurs in a match disputed by one of the Brazilian clubs against one of the Pernambuco’s head clubs, a positive constant is added to design the relation between them in terms of map position and their impact (contribution) in regards to the violence, defined by the size of each node. The larger the number of hooligan occurrences is for a specific match, and the bigger the number of matches with at least one hooligan incident, the bigger the nodes’ size and the smaller the edges connecting the head with their related opponent. Therefore, this representation indicates a distance-based relation, i.e., close heads and clubs in the network visualization, indicating a considerable grade of rivalry. Suppose there is a connection (an edge) between a head and a club. In that case, they are direct enemies when both the head and the club are inside the same circle cluster or indirect enemies if portrayed in different cluster circles.

**Figure 4:**
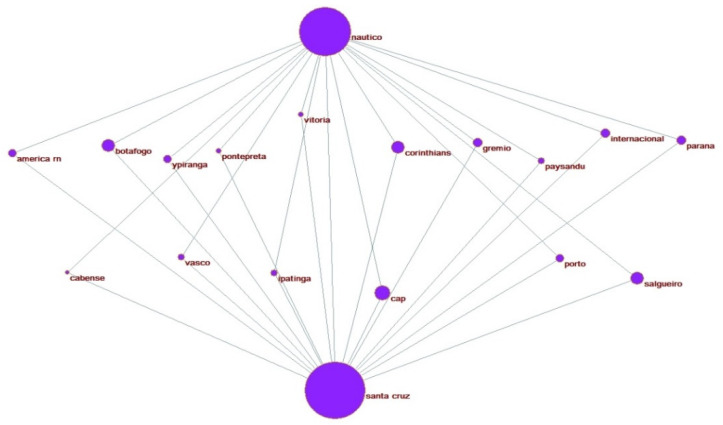
Coup affinity between Náutico and Santa Cruz.

**Figure 5:**
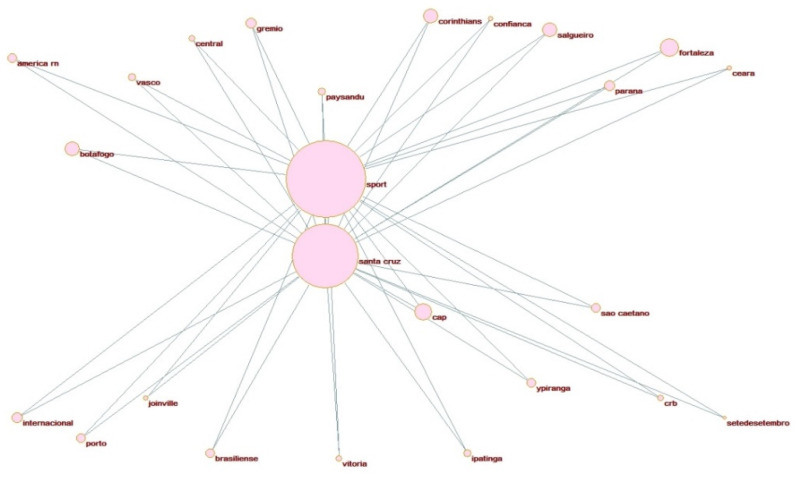
Clash affinity between Sport Recife and Santa Cruz.

**Figure 6:**
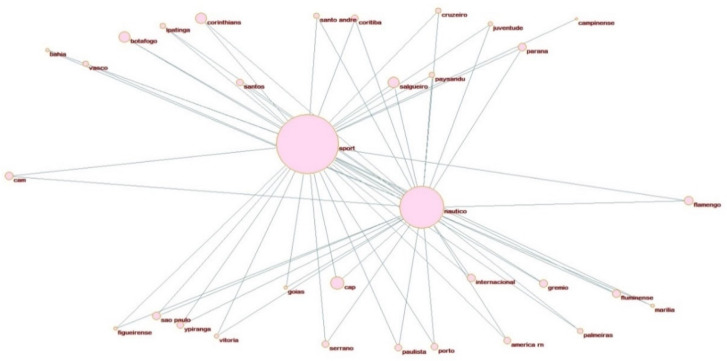
Clash affinity between Sport Recife and Náutico Capibaribe.

The same reasoning applies to the visualizations of Figure 4, Figure 5 and Figure 6 for specific affinity clusters. We considered all clubs with incidents of violence instead of the declared allies with occurrences only (in Table A1). A total of 72 clubs had at least one incident of violence with at least one of the head clubs in the past ten years of data. They are represented in this network. Three clusters can be observed in this representation. The clusters’ nodes represent each of Recife’s football clubs’ hostile interaction with other Brazilian clubs, i.e., their direct adversaries.

The most intense hostility association is held by the interaction between Sport Recife and Santa Cruz FC, which present about 19% of the total number of hooligan incidents recorded (see Table A2 in the Appendix A for the information about the number of hooligan occurrences, relevance score, and frequency of each club). The traditional rivalry between these clubs justifies this. Whenever a match is set at national or state levels to compete with each other, the media typifies this confrontation as the “crowd derby”, for which the greatest number of crimes and violence by fans is observed.

One important measure elicited in the network analysis is each interaction edge’s relevance. This information is provided in Table A2 in the Appendix A and has a significant meaning in evaluating the randomness of the path interaction [46]. The bigger this statistic, the more chances a violent behavior related to the analyzed club has to co-occur mainly with their cluster head. In this case, the clubs Fortaleza and Salgueiro (associated with Sport Recife) and Vera-Cruz (associated with Santa Cruz FC) are the most prone to violence with their heads, suggesting the matches received special attention from the public authorities in that specific year. Interesting information can be extracted from the network visualization related to the number of mutual enemies shared by each combination of heads. Despite the distance between Sport Recife and Náutico Capibaribe in the map visualization (indicating a smaller grade of rivalry), they have the most significant number of shared enemies compared to Santa Cruz FC: 31 enemies in total are shared. A possible explanation is that both SC Recife and Náutico Capibaribe shared the same national and state championship participation for most of the analyzed years. In contrast, Santa Cruz FC confronted different clubs from lower club divisions.

Lastly, two instances of the simultaneous affinities in Table 1 are designed in Figure 3, Figure 4 and Figure 5. These visualizations can provide additional information to support better public safety policies and strategies to identify (and prevent) the most relevant hooligan confrontations. Figure 4, Figure 5 and Figure 6 illustrate the potential Coup affinity between Náutico Capibaribe and Santa Cruz FC and the Clash affinities between Sport Recife and Santa Cruz FC and the Sport Recife and Náutico Capibaribe. According to Table 3, the fan bases of Náutico and Santa Cruz present an undefined underlying relation. Nevertheless, we define Náutico Capibaribe and Santa Cruz FC as mutual friends in the instance of Figure 4 for this application. The closeness of the two nodes represents their rivalry. Therefore, Náutico Capibaribe and Santa Cruz FC are positioned distantly to each other to represent a slight grade of rivalry.

From this visualization, the fan bases of 16 national clubs compose a coup with the two heads, i.e., two friends with a mutual enemy, or, in terms of Bedouin, an enemy of my friend is my enemy. The clubs are: América FC-RN, Botafogo FR, Ypiranga FC, AA Ponte Preta, EC Vitória, SC Corinthians, Grêmio FC, Paysandu SC, SC Internacioal, Paraná Clube, AD Cabense, CR Vasco da Gama, Ipatinga FC, Club Athletico Paranaense, CA Porto, and Salgueiro AC.

Sport Club Recife and Santa Cruz FC and Sport Club Recife and Náutico Capibaribe are defined as mutual enemies in Figure 5 and Figure 6, positioned close to each other, indicating a high grade of rivalry according to their score of affinity relation. The interpretation is as follows: when the affinity test has $A_{\left(a,k\right)}\;$> $A_{\left(b,k\right)}$, the club k is allocated nearby the head *a* in the network and allocated nearby the head b otherwise. If both have equal affinity scores (or *p*-value), the club k is allocated with the same distance between both heads. Figure 5 and Figure 6 show 23 and 31 clubs composing hostile Clash affinities between Santa Cruz FC, Sport Recife, and Sport Recife and Náutico Capibaribe. Depending on the context and environment, the construction of these networks can provide a valuable tool for the allocation of resources.

## 6. Conclusions

This paper tested the Bedouin syndrome hypothesis on the assumed rivalry between Brazilian football organized fan bases. As a main result, the analysis could not find statistical support for the Bedouin syndrome in most football firms’ declared alliances, suggesting that public policies based on these declarations could be ineffective. Based on these results, we provide a more attractive hooligan network construction methodology that can be more appropriate for identifying critical matches for allocating police resources.

We provide a methodology to construct social interactions among disputing or collaborating groups based on network mapping and statistical analysis. The identification and visualization of significant grouping relations in the hooligan networks were highlighted by a general network and two specific instances of the proposed alliance and rivalry methodology (coup and clash affinities). The network construction can support better security strategies with the possibility of rapid and easy interpretation of complex social relationships to support better decisions regarding policing strategies and resource allocation. However, a significant limitation is that the analyses focus only on three “head” clubs from the state of Pernambuco in the northeast region of Brazil. We hope to address this limitation in future extensions of this approach to more extensive datasets in the near future.

Another essential prospect of the proposed methodology is the identification of potential conflicting matches based on trends and patterns in the time series of data, visually represented in the size of nodes, clusters scales, length of edges, and poisoning of edges, which can be associated with contextual, spatial, social, and economic characteristics of the urban area to produce more accurate predictions. In addition, a wide set of econometric tools might be applied to picture a more significant social frame of the hooligan behavior with the environment and social conditions. The work of Nepomuceno et al. [11] is an example of how variables such as gates (audience), the tournament phase or relevance of matches, and many other factors can be combined with understanding those networks for better public security support.

Crime is dynamic, and this is one important limitation in our analysis. The propensity for hooliganism, especially when we construct clusters as direct enemies related to the heads, is due to punctual circumstances that rarely repeat through time. This requires some subjective judgment and experience when the quantitative analysis fails to propose a coherent picture of reality. We expect this evaluation of hooliganism and the proposed methodology can provide an avenue for this direction and support police officers and vehicle allocation strategies based on the empirical networks of violence.

## Figures and Tables

**Table 1:** Underlying Relations and Potential Affinities for Organized Fan Bases.

Underlying Relation	Affinity	Definition
Alliance	Coup	Friends with mutual enemy
	Peer	Friends with mutual friend
Rivalry	Clash	Enemies with mutual enemies
	Split	Enemies with mutual friend

**Table 2:** Bedouin affinities.

Affinity	Definition	Bedouin Association
Coup	Friends with a mutual enemy	Friend of my enemy is my enemy
		Enemy of my friend is my enemy
		Enemy of my enemy is my friend
Peer	Friend with a mutual friend	Friend of my friend is my friend

**Table 3:** The relations among the three heads.

Heads		Mean	Std. Deviation	*p*-Value	Underlying Relation
Torcida Jovem (Sport Club Recife)	Torcida Inferno Coral (Santa Cruz FC)	14.96	24.32	0.018	Rivalry
Torcida Jovem (Sport Club Recife)	Torcida Fanáutico (Clube Náutico Capibaribe)	6.027	8.036	0.048	Rivalry
Torcida Inferno Coral (Santa Cruz FC)	Torcida Fanáutico (Clube Náutico Capibaribe)	3.714	4.817	0.477	Undefined

**Table 4:** The hypotheses test based on occurrences declared enemies and friends.

Head	Organized Fan Bases	Mean	Std. Deviation	*p*-Value
Torcida Jovem (Sport Club Recife)	Força Jovem e Mancha Negra (Vasco)	6	4.358899	0.13857
	Galoucura (Atlético-MG)	1.66	2.73252	0.30494
	Bamor (Bahia)	1.5	0.707107	0.16674
	**Fúria Jovem do Ypiranga (Ypiranga)**	**0.5**	**0.707107**	**0.06417**
	Máfia Vermelha (América—RN)	2.5	2.12132	0.45412
	**TUP (Palmeiras)**	**0.833**	**0.752773**	**0.00131**
	Torcida Jovem (Botafogo)	6.33	6.110101	0.18477
	Geral (Grêmio)	2	2	0.41571
	Cearamor (Ceará)	1.5	2.12132	0.34699
Torcida Inferno Coral (Santa Cruz FC)	Máfia Azul (Cruzeiro)	0	0	-
	Independente e Dragões da Real (São Paulo)	0	0	-
	Jovem Fla (Flamengo)	0	0	-
	**Camisa 12 (Internacional)**	**6**	**1.414214**	**0.076807**
	Remoçada (Remo)	4	2.82843	0.257332
	Facção Brasiliense (Brasiliense)	5.5	3.535534	0.123865
	TUP (Palmeiras)	0	0	-
	Torcida Jovem (Botafogo)	4	5.656854	0.37086
	Geral (Grêmio)	2	2.828427	0.45549
	Cearamor (Ceará)	1	1.414214	0.2074
Torcida Fanáutico (Clube Náutico Capibaribe)	**Máfia Azul (Cruzeiro)**	**0.5**	**0.707107**	**0.06417**
	Independente e Dragões da Real (São Paulo)	3.5	4.949747	0.3934
	Jovem Fla (Flamengo)	3	2.645751	0.34287
	Camisa 12 (Internacional)	2.75	2.06155	0.34222
	Remoçada (Remo)	0	0	-
	Facção Brasiliense (Brasiliense)	0	0	-
	Força Jovem e Mancha Negra (Vasco)	1	1.414214	0.2074
	**Galoucura (Atlético-MG)**	**1**	**0.707107**	**0.00458**
	Bamor (Bahia)	1	1.414214	0.2074
	Fúria Jovem do Ypiranga (Ypiranga)	1.75	1.258306	0.23578
	Máfia Vermelha (América-RN)	2.5	2.12132	0.45412

First group statistics: Mean = 2.28181800; Std. Deviation = 3.91970700; SEM = 0.21577277; *n* = 330.

## Data Availability

The data supporting this study’s findings are available from the corresponding author on request to thyago.nepomuceno@ufpe.br.

## Appendix A

**Table A1:** Set of declared enemies and friends for each head gang (Torcidas Organizadas).

Fanbase (Head)	Fanbase (Clubs)	Declared Allied (Heads)	Affinity (H_0_)
Torcida Jovem (Sport Club Recife)	Força Jovem and Mancha Negra (Vasco)	Santa Cruz	Coup
	Galoucura (Atlético-MG)	Santa Cruz	
	Bamor (Bahia)	Santa Cruz	
	Fúria Jovem do Ypiranga (Ypiranga)	Santa Cruz	
	Máfia Vermelha (América—RN)	Santa Cruz	
	TUP (Palmeiras)	Náutico Capibaribe	
	Torcida Jovem (Botafogo)	Náutico Capibaribe	
	Geral (Grêmio)	Náutico Capibaribe	
	Cearamor (Ceará)	Náutico Capibaribe	
Torcida Inferno Coral (Santa Cruz FC)	Máfia Azul (Cruzeiro)	Sport Recife	Coup
	Independente e Dragões da Real (São Paulo)	Sport Recife	
	Jovem Fla (Flamengo)	Sport Recife	
	Camisa 12 (Internacional)	Sport Recife	
	Remoçada (Remo)	Sport Recife	
	Facção Brasiliense (Brasiliense)	Sport Recife	
	TUP (Palmeiras)	Náutico Capibaribe	Peer or Coup
	Torcida Jovem (Botafogo)	Náutico Capibaribe	
	Geral (Grêmio)	Náutico Capibaribe	
	Cearamor (Ceará)	Náutico Capibaribe	
Torcida Fanáutico (Clube Náutico Capibaribe)	Máfia Azul (Cruzeiro)	Sport Recife	Coup
	Independente e Dragões da Real (São Paulo)	Sport Recife	
	Jovem Fla (Flamengo)	Sport Recife	
	Camisa 12 (Internacional)	Sport Recife	
	Remoçada (Remo)	Sport Recife	
	Facção Brasiliense (Brasiliense)	Sport Recife	
	Força Jovem e Mancha Negra (Vasco)	Santa Cruz	Peer or Coup
	Galoucura (Atlético-MG)	Santa Cruz	
	Bamor (Bahia)	Santa Cruz	
	Fúria Jovem do Ypiranga (Ypiranga)	Santa Cruz	
	Máfia Vermelha (América-RN)	Santa Cruz	

**Table A2:** Relevance and frequency from the hooligan network.

id	Club	Occurrences	Relevance Score	Frequency		
				Sport	Santa Cruz	Náutico
1	abc	2	1.4631	0	2	0
2	alecrim	2	1.4631	0	2	0
3	america rn	14	0.0844	5	5	4
4	araripina	1	1.4865	1	0	0
5	asa	3	1.4865	3	0	0
6	avai	1	1.4631	0	1	0
7	bahia	4	0.6434	3	0	1
8	belojardim	5	0.2224	3	0	2
9	boaesporte	3	1.1721	0	0	3
10	botafogo	29	0.6207	19	4	6
11	bragantino	1	1.4865	1	0	0
12	brasiliense	4	1.324	1	3	0
13	cabense	3	1.3037	0	3	0
14	cam	17	0.9294	14	0	3
15	campinense	2	1.4865	2	0	0
16	cap	7	1.3959	3	1	3
17	ceara	4	0.7897	3	1	0
18	central	6	0.7897	5	1	0
19	chagrande	1	1.4631	0	1	0
20	confianca	4	0.7897	3	1	0
21	corinthians	25	0.1773	15	1	9
22	coritiba	13	0.7039	10	0	3
23	crb	1	1.2184	1	0	0
24	criciuma	1	1.4631	0	1	0
25	cruzeiro	7	0.7626	6	0	1
26	csa	1	1.4631	0	1	0
27	cuiaba	4	1.4631	0	4	0
28	figueirense	3	0.5653	1	0	2
29	flamengo	20	0.1813	11	0	9
30	fluminense	6	0.7862	3	0	3
31	fortaleza	41	1.2684	41	0	0
32	gama	6	1.4631	0	6	0
33	goias	1	0.7561	1	0	0
34	gremio	17	0.2102	6	2	9
35	guarani	1	1.4865	1	0	0
36	icasa	1	1.4631	0	1	0
37	imperatriz	1	1.4865	1	0	0
38	internacional	13	0.6569	5	5	3
39	ipatinga	8	0.2662	3	5	0
40	joinville	5	0.0252	3	2	0
41	juventude	6	1.256	6	0	0
42	luverdense	3	1.4631	0	3	0
43	marilia	2	0.388	1	0	1
44	nautico	336	0.6431	222	114	-
45	palmeiras	10	0.1874	6	0	4
46	parana	17	0.7171	12	3	2
47	paulista	1	1.1159	1	0	0
48	paysandu	9	0.9775	7	1	1
49	pesqueira	2	1.4865	2	0	0
50	pontepreta	2	1.1168	0	0	2
51	porto	9	0.2014	2	6	1
52	portuguesa	5	1.4865	5	0	0
53	potiguar	4	1.4631	0	4	0
54	pretolina	2	1.4631	0	2	0
55	remo	3	1.4631	0	3	0
56	riobranco	1	1.4631	0	1	0
57	salgueiro	33	1.1647	25	5	3
58	sampaiocorrea	3	1.4631	0	3	0
59	santa cruz	471	1.2581	357	-	114
60	santarita	1	1.4631	0	1	0
61	santo andre	5	0.2155	5	0	0
62	santos	12	0.9248	9	0	3
63	sao caetano	13	0.406	5	8	0
64	sao paulo	18	0.1878	11	0	7
65	sao raimundo	1	1.4865	1	0	0
66	serra talhada	5	1.4865	5	0	0
67	serrano	12	0.487	4	0	8
68	setedesetembro	3	0.1416	1	2	0
69	sport	579	1.208	-	357	222
70	treze	3	1.4631	0	3	0
71	vasco	20	0.9138	18	2	0
72	vera cruz	21	1.4631	0	21	0
73	vilanova	1	1.1721	0	0	1
74	vitoria	4	0.6178	1	2	1
75	ypiranga	11	0.8555	2	7	2
